# Prevalence of systemic immunoreactivity to *Aggregatibacter actinomycetemcomitans *leukotoxin in relation to the incidence of myocardial infarction

**DOI:** 10.1186/1471-2334-11-55

**Published:** 2011-03-01

**Authors:** Anders Johansson, Marie Eriksson, Ann-Marie Åhrén, Kurt Boman, Jan-Håkan Jansson, Göran Hallmans, Ingegerd Johansson

**Affiliations:** 1 Department of Odontology, Faculty of Medicine, Umeå University, Umeå, Sweden; 2 Department of Public Health and Clinical Medicine, Faculty of Medicine, Umeå University, Umeå, Sweden; 3 Department of Medicine-Geriatrics, Sweden

## Abstract

**Background:**

Chronic infections and associated inflammatory markers are suggested risk factors for cardiovascular disease (CVD). The proinflammatory cytokine, interleukin (IL)-1β, is suggested to play a role in the regulation of local inflammatory responses in both CVD and periodontitis. The leukotoxin from the periodontal pathogen *Aggregatibacter actinomycetemcomitans *has recently been shown to cause abundant secretion of IL-1β from macrophages. The aim of the present study was to compare the prevalence of systemic immunoreactivity to *A. actinomycetemcomitans *leukotoxin in myocardial infarction (MI) cases (n = 532) and matched controls (n = 1,000) in a population-based case and referents study in northern Sweden.

**Methods:**

Capacity to neutralize *A. actinomycetemcomitans *leukotoxin was analyzed in a bioassay with leukocytes, purified leukotoxin, and plasma. Plasma samples that inhibited lactate-dehydrogenase release from leukotoxin-lysed cells by ≥50% were classified as positive.

**Results:**

Neutralizing capacity against *A. actinomycetemcomitans *leukotoxin was detected in 53.3% of the plasma samples. The ability to neutralize leukotoxin was correlated to increasing age in men (n = 1,082) but not in women (n = 450). There was no correlation between presence of systemic leukotoxin-neutralization capacity and the incidence of MI, except for women (n = 146). Women with a low neutralizing capacity had a significantly higher incidence of MI than those who had a high neutralizing capacity.

**Conclusion:**

Systemic immunoreactivity against *A. actinomycetemcomitans *leukotoxin was found at a high prevalence in the analyzed population of adults from northern Sweden. The results from the present study do not support the hypothesis that systemic leukotoxin-neutralizing capacity can decrease the risk for MI.

## Background

Chronic inflammations, such as periodontitis, are suggested to be risk factors for the development of cardiovascular diseases [1]. It has been suggested that the total pathogenic burden from the oral cavity, and possibly also from the gut, correlates with disease markers of atherosclerosis [2]. Periodontitis is a bacteria-induced inflammatory condition that causes degradation of the tooth-supporting tissues, bone and connective tissue [3,4]. Bioactive molecules released from pathogenic microorganisms located in the subgingival biofilm cause imbalance in the inflammatory response, which results in loss of the tooth-supporting tissues [5]. For the host to maintain homeostasis within the periodontal tissues, the immune response system contributes to controlling the microbial colonization and invasion [6]. This immune response includes local and systemic production of antibodies induced by antigens from the microorganisms that are localized in this biofilm [7]. There are more than 700 different microbial species found in the oral cavity of humans [8]. A recent report using the pyrosequencing methodology to analyze the composition of the oral microbiota indicate a substantial increase in that number [9]. Among the different species found, *Aggregatibacter actinomycetemcomitans *is a bacterium associated with aggressive forms of periodontitis, and it produces a leukotoxin that specifically affects human leukocytes [10]. Individuals infected with a specific, highly leukotoxic clone (JP2) of this bacterium have a significantly increased risk for periodontitis [11]. The proinflammatory response induced by the leukotoxin is a cellular response associated with the pathogenesis of periodontitis [10,12,13] and atherosclerosis [14].

The proportion in a population that harbor *A. actinomycetemcomitans *varies depending on geographic origin and periodontal condition of the subjects [10]. It has been shown that systemic leukotoxin-neutralization is correlated to the presence of this bacterium in the oral subgingival biofilm [15-17]. Data from a previous study showed that women with systemic neutralizing capacity against *A. actinomycetemcomitans' *leukotoxin had a significantly decreased incidence of stroke [18]. This systemic neutralizing capacity has been shown to correlate (p-value < 0.001) to the presence of leukotoxin-specific antibodies, as well as to antibodies against whole *A. actinomyctemcomitans *bacteria [19]. We hypothesized that a virgin *A. actinomycetemcomitans *infection late in life might be a risk factor for stroke and contribute to the negative association between stroke and the presence of these neutralizing antibodies. The aim of the present study was to analyze if the presence of systemic immunoreactivity to *A. actinomycetemcomitans *leukotoxin also interferes with the incidence of a future myocardial infarction (MI).

## Methods

### Study population

The study population was derived from the Northern Sweden Health and Disease Study (NSHDS), which consists of three sub-cohorts: The Västerbotten Intervention Programme (VIP) [20], the WHO's Multinational Monitoring of Trends and Determinants in Cardiovascular Disease (MONICA) study in northern Sweden [21] and the Mammography Screening Project (MSP) [22]. Both VIP and MONICA are health examination programmes for CVD and diabetes. Participation rates were 59 and 77%, respectively. The VIP was designed to be as similar as possible to the MONICA study. In order to increase the number of female cases, participants in the MSP were included in sex specific analyses. The participation rate in the MSP was 85% in the screening phase, of which 57% donated blood samples. By December 31, 1999 approximately 73,000 unique subjects had been screened in these 3 sub-cohorts in NSHDS.

Incident cases and matching controls were identified through 13 years of follow-up (1985-1999) from the Västerbotten Intervention Program and the Multinational Monitoring of Trends and Determinants in Cardiovascular Disease (MONICA) study. Study participants were from the Västerbotten and Norrbotten counties in northern Sweden. Participants with a history of MI, stroke or cancer were excluded from this study. Participants were followed from baseline examination until first MI or death. There was an average time period of 4 years between the time of inclusion and the MI event.

Fatal and nonfatal cases of MI occurring from October 1, 1994 to December 31, 1999 were identified through the Northern Sweden Monica Incidence Registry [23]. Throughout the follow-up period, 532 incident first events of MI (cases) were identified. For each case, two controls were individually matched for age, sex and ± 4 months of case occurrence. Thus, the study population consisted of 1,532 participants, 532 cases (382 men and 156 women) and 1,000 sex and age-matched controls (706 men and 294 women), aged 30-77 years at baseline. This study population has previously been described in detail [24]. The study was approved by the Ethics Committee of Umeå University and was conducted in accordance with the Helsinki Declaration. All participants gave informed consent.

### Leukotoxin-neutralization assay

The *A. actinomycetemcomitans *leukotoxin-neutralizing capacity in the plasma samples was detected as a reduction of leukocyte damage and subsequent leakage of lactate dehydrogenase (LDH) upon exposure to purified leukotoxin, as described previously [18]. This assay quantifies the activity of the LDH enzyme and does not allow freezing and thawing of the supernatants. The neutralization assay also limits the possible influence from LDH present in the different plasma samples from the study population.

Briefly, human polymorphonuclear leukocytes (PMNs) were isolated from human peripheral blood as described previously [25]. The isolated PMNs were suspended in RPMI 1640 (Sigma-Aldrich, St Louis, MI, USA) with 10% fetal bovine serum (FBS) (Sigma-Aldrich) at a density of 3 × 10^6 ^cells/ml. The blood was taken from donors visiting the University Hospital blood bank in Umeå, Sweden. Informed written approval was given by all subjects, and authorization for the study was granted by the Human Studies Ethical Committee of Umeå University, Sweden (§67/3, dnr 03-019).

For detection of leukotoxin-neutralizing capacity, purified leukotoxin (450 ng/mL) [26] was mixed with each plasma sample (10%) in RPMI 1640. One hundred μl portions of the plasma-leukotoxin mixtures were added in duplicate to a 96 well culture plate (Nunc, Roskilde, Denmark) and incubated for 15 min at room temperature. Then 50 μl of PMN was added and the mixtures were incubated for 60 min at 37°C in 5% CO_2_. Activity of the released LDH into the culture supernatant was quantified as described previously [25]. Plasma samples that inhibited the leukotoxin-induced LDH release by ≥50% were classified as positive and were further analyzed in the assay diluted to 1% of the final volume. Plasma without leukotoxin-neutralization capacity was classified as "*null"*, plasma that neutralized leukotoxin at 10% plasma concentration but not at 1% was classified as "*low*", and plasma that neutralized the leukotoxin at both 10% and 1% concentrations was classified as "*high"*.

### Statistical anaylses

The Mantel-Haenszel χ^2^-test for trend was used to analyze the association between age and antibodies. To investigate if the presence of leukotoxin antibodies (categorized into null, low or high) affects the risk of having an MI, conditional logistic regression appropriate for the matched design, was used. A multivariable model was used to adjust for smoking, self-reported diabetes, systolic blood pressure and apoB/apoA1. Results are presented as p-values, odds ratios (ORs) and corresponding 95% confidence intervals (CIs). No correction for multiple testing was performed. SAS version 9.1 was used for the statistical analyses.

## Results

### Prevalence of leukotoxin-neutralizing capacity

**Figure 1:**
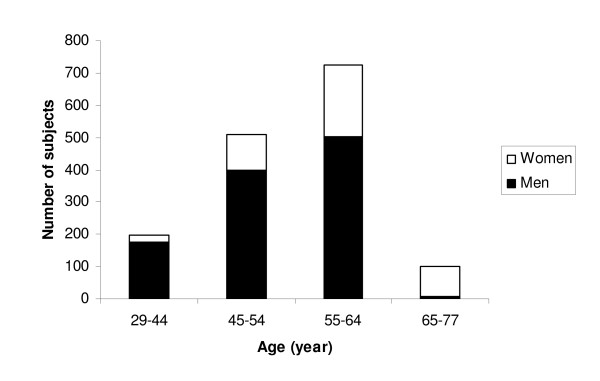
**Distribution of men and women in the age groups for the whole study cohort, i.e. including both cases and referents**.

The study population was classified into 4 different age groups, and the distribution in relation to age and gender is shown in Figure 1.

**Figure 2:**
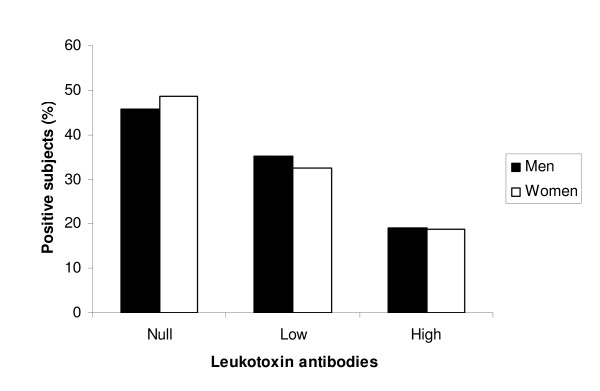
**Proportion of men and women with different systemic capacity to neutralize *A. actinomycetemcomitans *leukotoxin**.

Among the 1,532 analyzed plasma samples, 817 (53.3%) could neutralize *A. actinomycetemcomitans *leukotoxicity in the neutralization assay. Further dilution of the plasma samples resulted in loss of the capacity to neutralize leukotoxin in 526 of these samples, and they were classified as samples with low neutralizing capacity. The 291 samples that neutralized leukotoxin also at the higher dilution were classified as high. The distribution of the study population in relation to their systemic capacity to neutralize leukotoxin was 46.7% negative, 34.3% low and 19.9% high. There were no significant differences between men and woman in capacity to neutralize the leukotoxin (Figure 2).

### Prevalence and age

**Figure 3:**
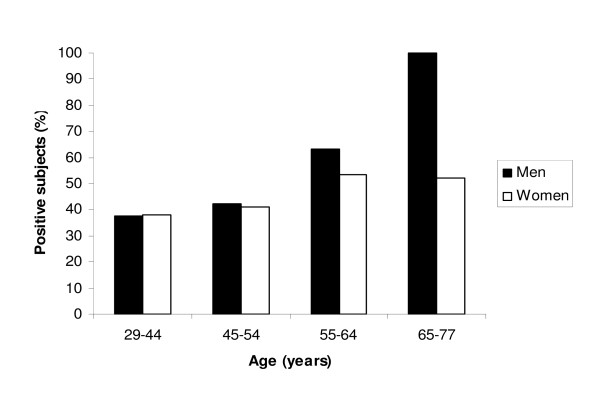
**Proportion of men and women (low + high) with systemic *A. actinomycetemcomitans *leukotoxin-neutralizing capacity in relation to age**. The distributions of men and women in the different groups were: 29-44 yr, 179 men and 21 women; 45-54 yr, 397 men and 114 women; 55-64 yr, 503 men and 221 women; and 65-77 yr, 6 men and 94 women.

The proportion of subjects with capacity to neutralize leukotoxin increased with increasing age (Figure 3). This age-related increase was significant for men (p-values ≤ 0.001) but not for women (p-values = 0.170). In order to avoid combinations with no or few observations, the two lowest age groups (29-44 and 45-54) were merged for women, and the two highest age groups (55-64 and 65-77) were merged for men in the formal analysis.

### Prevalence in relation to incidence of MI

Women with low capacity to neutralize leukotoxin had a significantly (p-value = 0.031) higher incidence of MI than women without the capacity to neutralize leukotoxin (Table 1). The odds ratio (OR) of having an MI in this group was 1. 8 (95% CI: 1.13-2.8). No other significant differences were seen between the incidence of MI and the different groups classified in relation to systemic leukotoxin neutralization and gender. After adjustments for other known risk-factors for MI (smoking, diabetes, systolic blood pressure and ApoB/ApoA1) there were no significant differences (Table 1).

**Table 1:** Proportion of controls and cases with plasma that neutralized leukotoxic activity.

*Men & Women*	Neg	Low	High
**Control**	47.1% (471)	33.3% (333)	19.6% (196)
**Case**	45.9% (244)	36.3% (193)	17.9% (95)

**OR**		1.1 (0.8-1.4)	0.9 (0.7-1.2)

**adjusted OR**		1.0 (0.7-1.4)	1.3 (0.9-2.0)

***Women***	**Neg**	**Low**	**High**

**Control**	51.7% (152)	28.6% (84)	19.7% (58)
**Case**	42.9% (67)	39.7%*(62)	17.3% (27)

**OR**		1.8* (1.1-2.8)	1.0 (0.8-1.3)

**adjusted OR^#^**		1.2 (0.5-2.9)	1.4 (0.5-4.3)

***Men***	**Neg**	**Low**	**High**

**Control**	45.2% (319)	35.3% (249)	19.5% (138)
**Case**	47.1% (177)	34.8% (131)	18.1% (68)

**OR**		0.9 (0.7-1.2)	0.8 (0.6-1.2)

**adjusted OR^#^**		1.0 (0.7-1.4)	1.3 (0.9-2.1)

Significant differences between controls and cases (*), number of subjects (n) and the odds ratio (OR) of having an MI compared with the antibody-negative group (null) are indicated.

^#^) Adjusted for smoking, self-reported diabetes, systolic blood pressure and ApoB/ApoA1

## Discussion

Results from the present study showed that 53.3% of the plasma samples from a Swedish adult cohort of 1,532 subjects had the capacity to neutralize *A. actinomycetemcomitans *leukotoxin. It has recently been demonstrated that this leukotoxin-neutralizing capacity correlates with the presence of leukotoxin-specific antibodies [19]. The high prevalence of subjects with this leukotoxin-neutralizing capacity was not expected, however, in line with results from some previous studies [19,27]. Both of these studies were based on Swedish study populations from a similar age group as examined in the present study. In one of these reports the study population consisted of a total of 197 subjects from a case control study of myocardial infarction and matched healthy controls, and the prevalence of systemic leukotoxin-neutralizing capacity was 57% without significant differences between cases and controls [19,28]. The other study consisted of 50 subjects with periodontitis and 41 healthy referents, and in this population the prevalence of systemic leukotoxin-neutralizing capacity was 45% without significant differences between the two groups [27]. Another study showed lower prevalence (15.2%) of leukotoxin-neutralizing capacity [18]. In this study [18] a target cell line (HL-60) with lower sensitivity to leukotoxin than the PMNs was used in the neutralization assay [29], which resulted in a need for enhanced leukotoxin concentration to obtain cell lyses and subsequently more antibodies for neutralization. This difference in leukotoxin sensitivity makes this assay with PMNs more sensitive to detect leukotoxin neutralization than the previous used assay with HL-60 cells [15]. The proportion of samples (19.9%) with high leukotoxin-neutralizing capacity in the present study might be comparable with the results from Johansson et al., 2005 [18].

We have previously shown that the systemic leukotoxin-neutralizing capacity correlated to a decreased incidence of stroke in women [18]. The mechanisms behind this phenomenon are not known. We speculate that a virgin infection with *A. actinomycetemcomitans *in middle-aged and elderly subjects might be a risk factor for stroke and that the capacity to neutralize leukotoxin might be protective. The leukotoxin has been shown to induce a rapid proinflammatory reaction in human macrophages, already at a ratio of 1 bacterium/macrophage [13], and therefore the leukotoxin is a possible risk factor in atherosclerosis. The common etiology of both stroke and MI with inflammatory processes and atherosclerosis [14], indicates that the presence of systemic leukotoxin-neutralizing capacity also might interfere with the incidence of MI. The results of the present study showed that systemic presence of leukotoxin-neutralizing capacity did not affect the incidence of MI, except for women classified as low for this neutralizing capacity. Our main finding refutes the hypothesis that systemic immunoreactivity to leukotoxin has a protective effect against MI. The significant association found for women might be an effect of multiple testing or a type-1 error, but further studies on this finding are warranted to confirm this association.

The periodontal status of the analyzed subjects is not known, but it could be expected to be in line with a similar recently examined Swedish population [28]. In that population a correlation between MI and periodontitis was observed. In addition, both periodontitis and MI correlated to the presence of systemic immunoreactivity against *Porphyromonas gingivalis *but not against *A. actinomycetemcomitans *[19,28].

The proportion of subjects with systemic neutralizing capacity to leukotoxin increased with increasing age, significantly for men but not for women. This age-related increase is in line with previous findings [19] and indicates that a virgin infection with *A. actinomycetemcomitans *can take place late in life. A virgin *A. actinomycetemcomitans *infection might decrease the risk for stroke in middle-aged and elderly subjects without systemic leukotoxin-neutralizing capacity [18]. Even though the role of leukotoxin-neutralizing antibodies in the pathogenesis of periodontal disease is unknown [7], the antibodies might help mitigate the systemic effects of *A. actinomycetemcomitans *infections. The leukotoxin produced by *A. actinomycetemcomitans *is a unique virulence factor with the capacity to cause a rapid proinflammatory reaction [10]. However, to fully investigate a potential role of leukotoxin in the pathogenesis of stroke, the presence of systemic leukotoxin antibodies has to be analyzed both before and after the disease incidence. We therefore still look at the results that showed a negative correlation between systemic leukotoxin antibodies and stroke as preliminary [18].

## Conclusions

The results from the present study do not support the hypothesis that systemic leukotoxin-neutralizing capacity can decrease the risk for MI. In addition, the prevalence of systemic *A. actinomycetemcomitans *leukotoxin-neutralizing capacity is high (53.3%) in adults from northern Sweden. The prevalence of leukotoxin-neutralizing capacity increased with increasing age, significantly for men but not for women.

## Competing interests

The authors declare that they have no competing interests.

## Authors' contributions

AJ conceptualized the study, conducted the analyses, and wrote the first draft of the manuscript. A-MÅ and AJ performed the analyses and made a first draft of the result calculations. J-HJ and KB planned and supervised data collection. GH and IJ coordinated data collection, and ME performed the statistical calculations. All approved the final version of the submitted manuscript.

## Pre-publication history

The pre-publication history for this paper can be accessed here:

http://www.biomedcentral.com/1471-2334/11/55/prepub
